# Release of Ciprofloxacin-HCl and Dexamethasone Phosphate by Hyaluronic Acid Containing Silicone Polymers

**DOI:** 10.3390/ma5040684

**Published:** 2012-04-19

**Authors:** Darrene Nguyen, Alex Hui, Andrea Weeks, Miriam Heynen, Elizabeth Joyce, Heather Sheardown, Lyndon Jones

**Affiliations:** 1 Centre for Contact Lens Research, School of Optometry, University of Waterloo, 200 University Avenue West, Waterloo, ON N2L 3G1, Canada; E-Mails: d8nguyen@uwaterloo.ca (D.N.); heynen@uwaterloo.ca (M.H.); emartell@uwaterloo.ca (E.J.); sheardow@univmail.cis.mcmaster.ca (H.S.); lwjones@uwaterloo.ca (L.J.); 2 School of Biomedical Engineering, McMaster University, 1280 Main Street West, Hamilton, ON L8S 4L7, Canada; E-Mail: weeksak@univmail.cis.mcmaster.ca; 3 Department of Chemical Engineering, McMaster University, 1280 Main Street West, Hamilton, ON L8S 4L7, Canada

**Keywords:** hyaluronic acid, silicone containing hydrogel, ciprofloxacin-HCl, dexamethasone phosphate, drug delivery

## Abstract

The purpose of this study was to determine the effect of the covalent incorporation of hyaluronic acid (HA) into conventional hydrogel and hydrogels containing silicone as models for contact lens materials on the uptake and release of the fluoroquinolone antibiotic ciprofloxacin and the anti-inflammatory steroid dexamethasone phosphate. A 3 mg/mL ciprofloxacin solution (0.3% w/v) and a 1 mg/mL dexamethasone phosphate solution (0.1%) was prepared in borate buffered saline. Three hydrogel material samples (pHEMA; pHEMA TRIS; DMAA TRIS) were prepared with and without the covalent incorporation of HA of molecular weight (MW) 35 or 132 kDa. Hydrogel discs were punched from a sheet of material with a uniform diameter of 5 mm. Uptake kinetics were evaluated at room temperature by soaking the discs for 24 h. Release kinetics were evaluated by placing the drug-loaded discs in saline at 34 °C in a shaking water bath. At various time points over 6–7 days, aliquots of the release medium were assayed for drug amounts. The majority of the materials tested released sufficient drug to be clinically relevant in an ophthalmic application, reaching desired concentrations for antibiotic or anti-inflammatory activity in solution. Overall, the silicone-based hydrogels (pHEMA TRIS and DMAA TRIS), released lower amounts of drug than the conventional pHEMA material (*p* < 0.001). Materials with HA MW132 released more ciprofloxacin compared to materials with HA MW35 and lenses without HA (*p* < 0.02). Some HA-based materials were still releasing the drug after 6 days.

## 1. Introduction

Topical ophthalmic solutions, or eye drops, are used to deliver drugs to the anterior segment of the eye and account for approximately 90% of ophthalmic medications [1]. The use of eye drops as a drug delivery system to the eye has numerous advantages. Eye drops are simple and convenient for patient self-administration, and efficacious in treatment of medical conditions, since topical application delivers a high concentration of drug to the target site. However, a major disadvantage is low drug bioavailability. An instilled dose is in contact with the ocular surface for approximately 2 minutes, and only about 5% is absorbed into the eye [2], with most of it being lost to nasolacrimal drainage, spillage onto the cheek and conjunctival absorption. Blinking spreads the drop over the ocular surface and the conjunctiva becomes a major route of non-corneal absorption due to its larger surface area, vascularization and permeability compared to the cornea. Systemic absorption of the drug occurs through the conjunctiva and nasal mucosa in the nasolacrimal duct and may cause unwanted, adverse systemic effects. Rapid clearance from the ocular surface also means the drug has very little time to penetrate the cornea. The most effective corneal barrier is the epithelium, which is relatively impermeable due to the presence of tight junctions in the superficial layers, allowing only select small molecules to pass through, while largely excluding larger macromolecules. Since drug bioavailability and corneal penetration is low, topical ophthalmic solutions are formulated at higher concentrations and instilled multiple times a day in order to deliver an effective dose and equally importantly, prevent sub optimal dosing. Eye drops thus provide pulse delivery, with an initial transient overdose followed by a short period at therapeutic dose and then a prolonged period of sub-therapeutic dosing, necessitating the need for frequent instillation [3]. Patient compliance then becomes problematic, as the complexity of the dosing schedule increases. In addition, as the concentration of drugs increase, discomfort and irritation experienced by the patient also increases, further decreasing compliance [4]. The efficacy of the treatment regimen is further limited by problems with appropriate administration [5], and typically the same drop volume is not administered every time such that the drug is delivered at a variable rate. 

A potential, more efficient alternative to eye drops is a controlled release drug delivery system such as a drug-delivering soft contact lens or punctal plug. Soft contact lenses were first evaluated as a vehicle for ophthalmic drug delivery by Sedlacek in 1965 [6]. The objective was to provide slow, sustained drug release to the ocular surface, thus increasing bioavailability and penetration of the drug through the cornea. Intraocular absorption of a drug has been found to increase ten times when a drug-soaked contact lens was worn compared to instillation of eye drops [7], and this effect of improved bioavailability and ocular penetration has also been found with aminoglycosides and fluoroquinolone antibiotics [8]. Other studies comparing drug delivery with a soft contact lens to subconjunctival injection found that higher drug penetration was achieved with soft contact lenses and was greater with higher water content lenses [9]. Drug loading into a hydrogel soft contact lens has been typically achieved by soaking the lens in drug solution or applying drops while the lens is being worn [10]. Either method improves drug retention on the ocular surface and corneal penetration, while simultaneously minimizing systemic absorption, although release kinetics typically mimic uptake kinetics. In these cases, the lenses act like drug reservoirs, because their highly porous structure and high water content enable diffusion of drugs into and out of the lens [11]. Limited mixing and exchange of the tear film underneath soft contact lenses also helps retain drug on the ocular surface longer than eye drops [12]. Improved corneal penetration suggests that lower concentration drug solutions could be used to attain therapeutic effects [9], which would also decrease the likelihood of an ocular adverse reaction. In addition, controlled delivery systems such as drug containing contact lenses have the potential to improve patient compliance because the treatment regimen would be simplified [13] and patients would have clear, comfortable vision since they can wear their refractive correction while being treated [10]. However, using commercial materials without any kind of modification has led to an inability to sustain drug release for more than a day and with a release profile that is characterized by a significant initial burst, followed by subtherapeutic dosing [3], suggesting that modification of the lens materials is necessary in order to achieved sustained, controlled drug release. 

Hyaluronic acid (HA), discovered by Meyer and Palmer in 1934 [14], is a high molecular weight, non-sulfated [15,16,17] glycosaminoglycan that occurs naturally throughout the body. It is mainly found in synovial fluid, vitreous, the umbilical cord, and loose connective tissues, such as the dermis. HA consists of unbranched, linear chains composed of repeating units of glucoronic acid and N-acetylglucosamine. These molecules aggregate to form a mesh-like sheet, which in larger numbers form molecular networks. HA is highly attractive to water, having 10–15 hydrogen bond accepting atoms per disaccharide unit [18], and swells to a much larger volume than that of its unhydrated state [19]. In solution, HA has an expanded random coil configuration and the chains are entangled [20]. The molecular weight of HA depends on its source and affects its physicochemical properties and physiological role [14]. HA networks regulate water movement, and transport and exclusion of molecules through its mesh-like structure [21]. HA plays an important structural role in connective tissues and acts as a lubricant and shock absorber in joints to protect against mechanical damage [20]. HA also has roles in cellular activities during embryonic development and morphogenesis, cell adhesion, inflammation, and tissue regeneration and repair [18]. 

HA is a biocompatible, non-immunogenic, and biodegradable biomaterial. The medical applications of HA were pioneered by Balazs, who was the first to create non-inflammatory preparations of HA on a commercial scale to replace vitreous and aqueous humor in ocular surgery [22]. These preparations were also found to protect the corneal endothelium from damage during surgery. Commercial preparations containing HA have also been used as a viscosupplement in treating osteoarthritis, in surgery to prevent post-operative adhesions, and studied in wound healing, tissue engineering, and drug delivery [14]. HA solutions were found to have positive effects in ocular drug delivery studies. Studies from the late 1980s showed that adding HA to pilocarpine solutions improved bioavailability by increasing drug solution viscosity, so that ocular contact time was prolonged [23,24,25,26] and similar results were found for many other drugs [18]. It is also thought that mucoadhesion might play a role in the ability of HA to increase precorneal residence of a drug, because HA binds to the mucin layer on the corneal epithelium [18,20]. We have previously developed a series of conventional and silicone hydrogel like materials containing HA [27,28]. When covalently attached to the polymer matrix, the presence of HA was found to change properties such as water uptake and protein interactions. Others have shown the presence of poly(ethylene glycol) in silicone materials can alter release kinetics and protect protein activity [29]. Therefore the presence of HA in these materials may facilitate drug release from silicone and silicone hydrogel like materials. While there have been numerous studies using HA as a release matrix, to-date there have been no studies using HA as an excipient to help facilitate release. We hypothesize based on the previous results with PEG that the presence of HA covalently incorporated into the hydrogel matrix can be used to control drug release.

Ciprofloxacin (CF) is a broad-spectrum fluoroquinolone antibiotic available as a topical ophthalmic solution or ointment to treat bacterial conjunctivitis and bacterial corneal ulcers [30]. It is bactericidal to gram-negative and gram-positive bacteria by inhibiting the enzymes DNA gyrase (topoisomerase II) and topoisomerase IV that maintains the superhelical DNA structure during DNA synthesis. The treatment regimens for these infections are quite frequent due to inherent inefficiency of eye drops and the short CF residence time [31]. Treatment of even relatively mild bacterial conjunctivitis, for example, requires one to two drops four times a day, while treatment of sight threatening bacterial ulcers may have the drop instilled every fifteen minutes until further notice [30]. While adverse reactions are rare, the most frequently reported ones are localized burning, itching, foreign body sensation, formation of white precipitates associated with long-term use, redness, swelling, and photophobia. 

Dexamethasone phosphate (DXP) is a synthetic glucocorticoid, designed to enhance the anti-inflammatory properties of natural steroids produced by the adrenal glands. It is used ophthalmically to treat signs and symptoms of inflammation within the eye, including but not limited to redness, edema, heat and pain [32]. Prolonged treatment of the eye with dexamethasone has been linked to an increase in cataract formation, as well as an increase in intraocular pressure, potentially leading to glaucoma [33]. Regular monitoring of the intraocular pressure of patients being treated with dexamethasone is warranted. Use of dexamethasone or any steroid on the eye has the potential to exacerbate infection, and prolong wound healing times, thus it is used with caution [34]. 

The purpose of this study was to measure uptake and release of ciprofloxacin and dexamethasone phosphate from HA-based conventional and silicone hydrogel (SH) model soft lens materials to evaluate the potential of these materials as drug delivery devices.

## 2. Results and Discussion 

### 2.1. Ciprofloxacin-HCl Release from HA Materials

**Figure 1:**
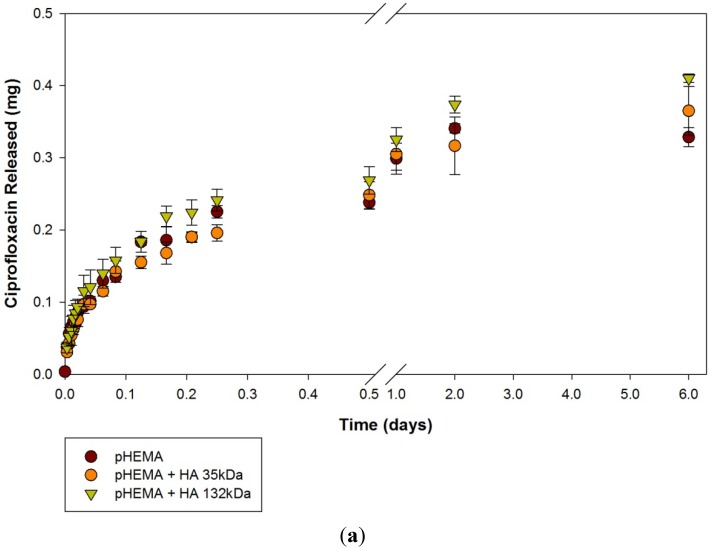

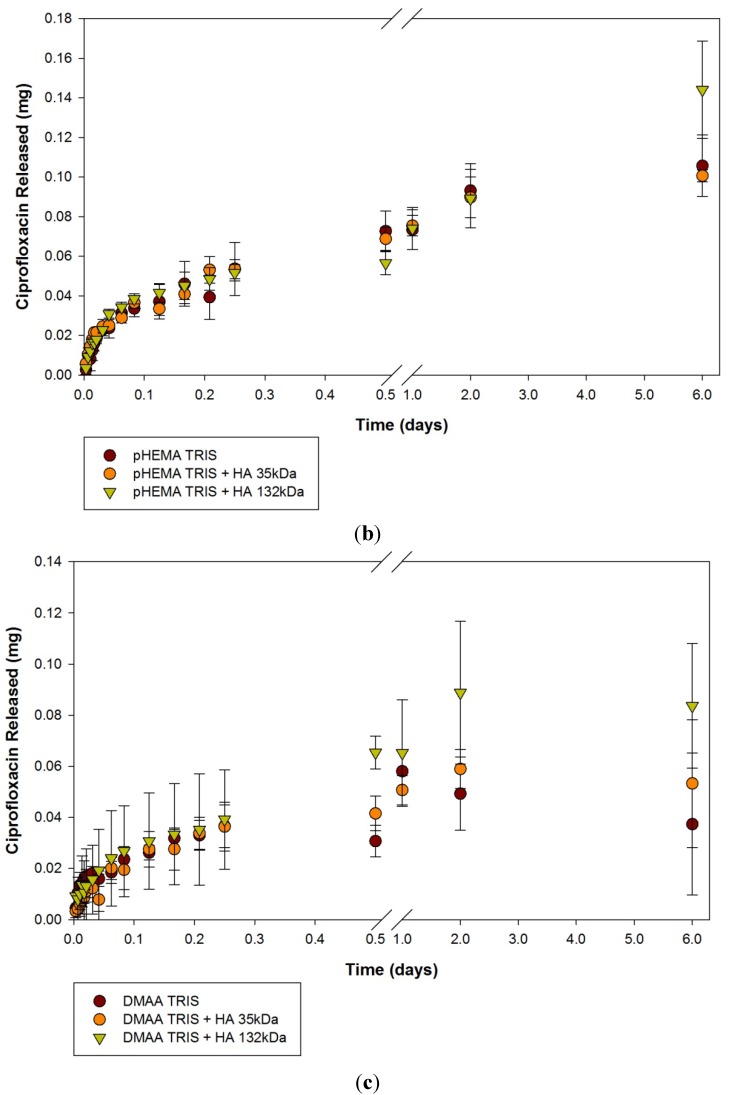
Ciprofloxacin Release curves from (**a**) pHEMA, (**b**) pHEMA TRIS and (**c**) DMAA TRIS materials. Values plotted are means ± SD. HA 35 kDa = 35 kDa Hyaluronic Acid. HA 132 kDa = Hyaluronic Acid 132 kDa.

Release curves over a 6-day period for ciprofloxacin loaded materials are presented in Figure 1(a–c). The discs are divided into three groups based on hydrogel material, either poly(2-hydroxyethyl methacrylate) (pHEMA), poly(2-hydroxyethylmethacrylate) tris(trimethylsiloxy)silylpropyl methacrylate (pHEMA TRIS) or N,N-dimethylacrylamide tris(trimethylsiloxy)silylpropyl methacrylate (DMAA TRIS). The release profiles from all three model materials showed similar patterns, with rapid initial release that slowed towards, but did not reach, a plateau. Ciprofloxacin release could be sustained for up to 6 days depending on the hydrogel type. pHEMA control discs and pHEMA + HA 132 kDa discs released roughly similar amounts of ciprofloxacin, while pHEMA + HA 35 kDa discs showed slower release kinetics, with less drug being released in the first 12 h (Figure 1a). After 12 h, the highest ciprofloxacin release was seen from pHEMA controls discs, followed by pHEMA + HA 132kDa discs, then pHEMA + HA 35 kDa discs. At the end of 6 days, however, pHEMA + HA 132kDa discs released the highest total amount of ciprofloxacin (361 ± 5 µg), and pHEMA discs released the least (328 ± 13 µg). 

For the first 6 h, pHEMA TRIS and pHEMA TRIS + HA 132 kDa discs released roughly the same amount of ciprofloxacin, and pHEMA TRIS + HA 35 kDa discs released the least (Figure 1b). This seems to suggest that in these materials, higher molecular weight of HA interacts with the hydrophilic drug which led to a slower release, at least initially. After 6 h, pHEMA TRIS control discs showed slightly more ciprofloxacin release than pHEMA TRIS + HA 132 kDa discs, but at the end of 6 days, pHEMA TRIS + HA 132 kDa discs released the highest amount of ciprofloxacin (133 ± 22 µg), which was almost double what pHEMA TRIS + HA 35 kDa discs released (85 ± 0.4 µg) despite that fact that our previous studies have shown that the materials showed similar levels of water uptake. Therefore, the higher amounts of drug present in the materials containing 132 kDa HA are presumably due to interactions between the incorporated HA and the drug. 

Similar to the results with pHEMA TRIS, in the first hour, DMAA TRIS control discs and DMAA TRIS + HA 35 kDa discs released roughly similar amounts of ciprofloxacin, while pHEMA + HA 132 kDa discs released more (Figure 1c). After one hour, pHEMA + HA 132 kDa discs continued to show the highest release, (86 ± 22 µg), and DMAA TRIS + HA 35 kDa discs began to show higher ciprofloxacin release than DMAA TRIS control discs, the difference increasing as time went on. These results clearly suggest that the presence of HA in the materials in general leads to increased uptake of the ciprofloxacin and hence, greater release. The lowest ciprofloxacin release was from DMAA TRIS control discs, at 49 ± 3 µg. 

However, in all cases, the kinetics of release was similar. All discs showed a similar and expected pattern of drug release, with initially rapid kinetics followed by decreasing release over time, which suggests that there were no interactions between the HA and the drug. This type of square root time release profile has been observed in several other studies that looked at ciprofloxacin release from soft contact lenses over a period of 24 h or less [8,23,35,36,37]. Unlike the commercially available contact lenses in those studies, most of these HA-based model lens materials showed measurable ciprofloxacin release for up to six days. Some authors [38] also found that adding HA to their hydrogel resulted in sustained ciprofloxacin release, which increased as the content of HA was increased. However, in these materials, the HA formed the gel. In these materials, a low degree of methacrylation of the HA ensured that the HA was incorporated along the polymer backbone. The thickness of these materials may have played a role in the extended release profiles observed compared with the swollen contact lenses previously examined, although more than 7 fold increase in the release time would not be expected based on the increased thickness of the materials. As well, there is some linearity in the first 12 h of ciprofloxacin release that is an improvement over the release profiles of commercial contact lenses that release most of their drug within 24 h. 

The average total mass of ciprofloxacin released over the 6 day period from each of the samples is presented in Table 1. Ciprofloxacin release was higher for materials with added 132 kDa HA than 35 kDa HA, but this difference was also not found to be statistically significant (*p* > 0.05). Studies by other authors did find that different molecular weights of HA affected drug delivery [26]. They compared high- and low-molecular-weight fractions of HA in pilocarpine solutions and found that adding higher MW HA produced better ocular retention times. In a volume of 2 mL, all discs released enough ciprofloxacin to meet the minimum inhibitory concentration (MIC_90_) previously demonstrated for many common ocular pathogens [35]. 

**Table 1:** Total mass (µg) of ciprofloxacin released from all model lens materials.

Modification	None (control)		HA 35		HA 132	
Material	mean	SD	mean	SD	mean	SD
pHEMA	328	13	337	33	361	5
pHEMA TRIS	99	12	85	0.4	133	22
DMAA TRIS	48	3	68	5	86	22

The rate of drug release is important because in an ophthalmic application, the tear film is drained and replenished at a regular rate. While these materials are thicker than a typical contact lens and therefore cannot be taken as representative of lens materials, for use in front of the eye application, the delivery system must be able to maintain effective concentrations of drug throughout the prescribed wearing period, as unabsorbed drug is eliminated from the ocular surface. Slower release rates enable more drug to be absorbed into the eye rather than into the systemic system [10]. All discs released the highest concentration of ciprofloxacin during the first hour. In the second hour, the concentration of drug released decreased to at least half the rate and continued to decrease for the remainder of the testing period. Only pHEMA TRIS + 132 kDa HA discs released concentrations of ciprofloxacin per hour that met the MIC_90 _for up to 6 days. However, the systems were swollen in a relatively low concentration solution of the ciprofloxacin; swelling in higher concentrations of the drug could be used to increase drug release. 

**Figure 2:**
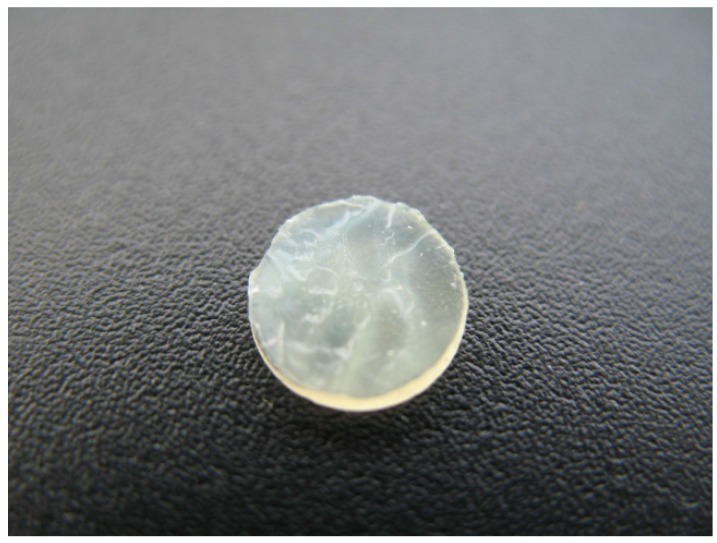
Ciprofloxacin precipitate film on the surfaces of pHEMA TRIS + HA 132 kDa disc.

**Figure 3:**
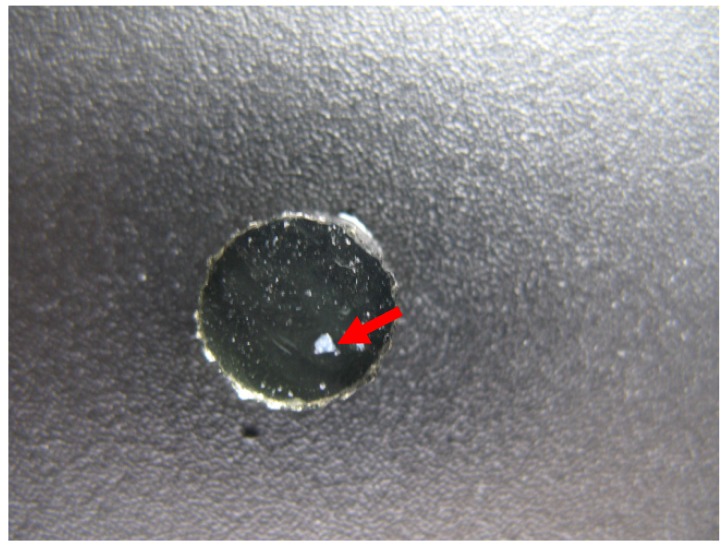
Ciprofloxacin precipitate on the surfaces of DMAA TRIS + HA 35 kDa disc.

Ciprofloxacin is not very soluble at physiological pH [35] so the discs were visually inspected for white precipitates. During the release phase, white precipitate films were seen on the surfaces on pHEMA TRIS + HA 132 kDa discs (Figure 2) and loose, white precipitate clumps were loosely attached to the surfaces of DMAA TRIS + HA 35 kDa discs (Figure 3). All other discs were clear of white precipitates. Ciprofloxacin precipitation does not require cessation of treatment [30], but it may interfere with the optical performance. Therefore, careful consideration of the material drug interaction is critical in the eventual usefulness of these types of devices and may warrant further investigation.

### 2.2. Dexamethasone Release from HA Materials

**Figure 4:**
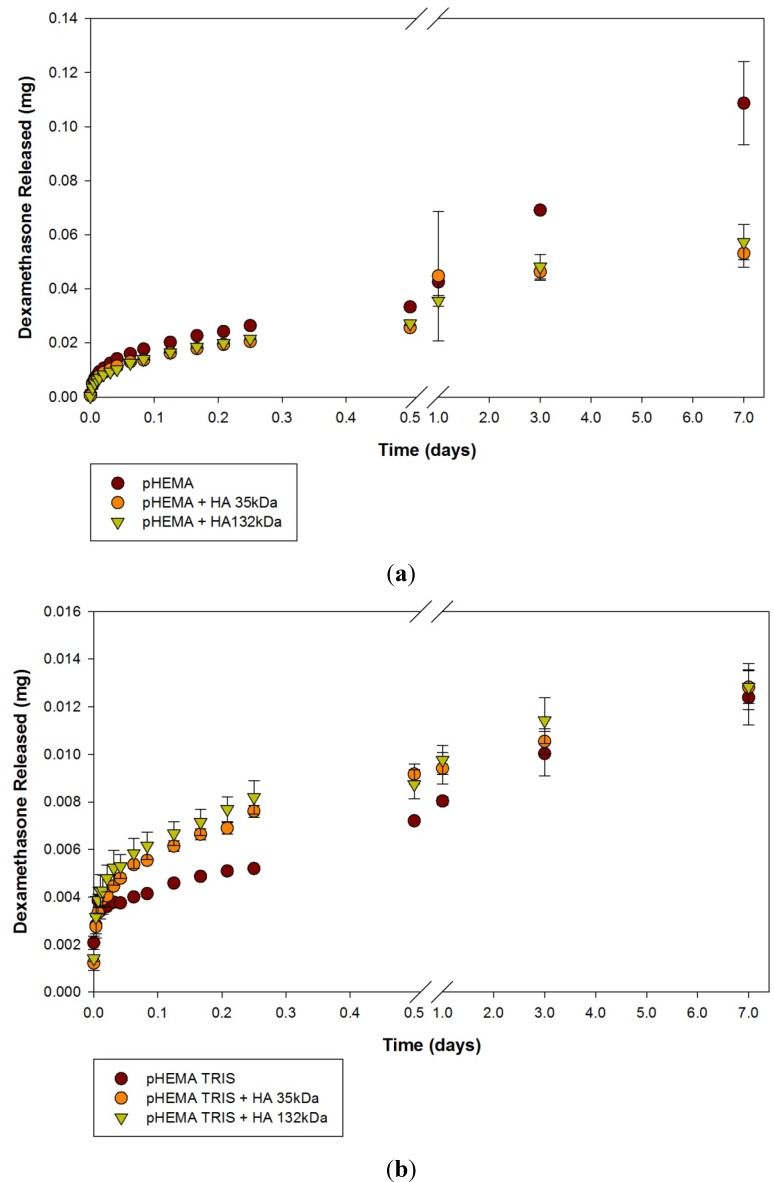

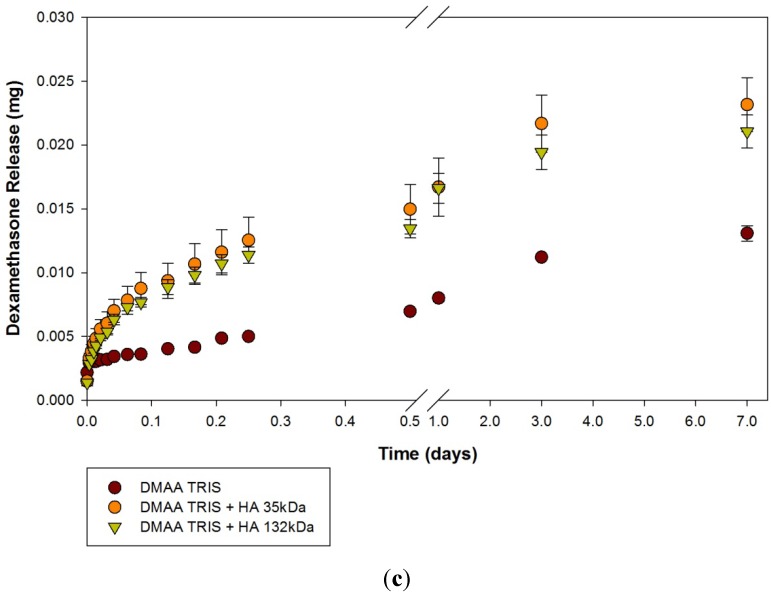
Dexamethasone phosphate release curves from (**a**) pHEMA, (**b**) pHEMA TRIS and (**c**) DMAA TRIS materials. Values plotted are means ± SD. HA 35 kDa = 35 kDa Hyaluronic Acid. HA 132 kDa = Hyaluronic Acid 132 kDa.

Release results for the dexamethasone phosphate loaded materials are presented in Figure 4(a–c). Again, the release curves are organized based on hydrogel material. Similar types of release patterns were seen, with the rate of drug release slowing over time, and moving toward a plateau. Sustained release of dexamethasone was seen with some materials for the 7 day monitoring period. pHEMA control materials released the largest amount of dexamethasone over the 7 day period (Figure 4a). While the pHEMA + HA 35 kDa and pHEMA + HA 132 kDa released less than the pHEMA control, the amount released was greater than from any other material, with no statistically significant difference between the two molecular weights of HA using pHEMA based discs (*p* > 0.05).

Similar results were seen between the two materials modified with the TRIS silicone component. For the first 6 h, the pHEMA TRIS + HA 35 kDa and pHEMA TRIS + HA 132 kDa model lenses released more dexamethasone and over a more prolonged period of time than the pHEMA TRIS control (Figure 4b), as did the DMAA TRIS + HA 35 kDA and DMAA TRIS + HA 132 kDa compared to the DMAA TRIS control. For pHEMA TRIS model lenses, all model lenses eventually released similar amounts of dexamethasone phosphate after 7 days (*p* > 0.05). For DMAA TRIS model lenses on the other hand, there was a statistically significant greater amount of drug released after 7 days for the HA modified materials versus the control.

All of the dexamethasone loaded materials released significant amounts of dexamethasone. All pHEMA materials released a significant amount of dexamethasone within 45 minutes. The incorporation of a more hydrophobic TRIS component led to slower release rates and generally lower amounts of released drug, although the latter is presumably due to lower loading with the lower swelling of the TRIS containing materials. The total amount of dexamethasone released from each model material is summarized in Table 2. The pHEMA based materials released statistically significantly more dexamethasone than the TRIS incorporated materials, although surprisingly release amounts from the pHEMA materials were not increased by the increased swelling that we have previously demonstrated accompanies the incorporation of HA. In DMAA TRIS, incorporation of HA led to a statistically greater amount of HA to be released (*p* < 0.002). The effect of HA was not significant for pHEMA TRIS based materials (*p* > 0.05).

**Table 2:** Total mass (µg) of dexamethasone phosphate released from all model lens materials.

Modification	None (control)		HA 35		HA 132	
Material	mean	SD	Mean	SD	mean	SD
pHEMA	109	15	53	5	57	6
pHEMA TRIS	12	1	13	0.7	13	0.9
DMAA TRIS	13	0.6	23	2	21	1

Previous work with contact lenses has shown that drug penetration and subsequent release depends on material properties as well as the properties of the drug and drug-loading solution [9,10]. For a given lens material, different drugs have different diffusion coefficients and different solubilities [10]. Drug uptake and release are affected by such lens properties as lens polymer, water content, three-dimensional structure and lens thickness. There is evidence that higher water content lenses show greater drug uptake in general [10,39], while some evidence suggests that lower water content lenses show longer release times [10]. Conventional hydrogels are generally considered more porous than SH hydrogels [40] so it is likely that the higher amounts of drug released from the pHEMA materials was the result of increased loading of the drug in these materials, presumably due to their higher water content. SH materials have a relatively low water content compared to conventional hydrogels and those containing TRIS take up less water [41], which might have resulted in lower drug uptake, and consequently, lower release than the conventional hydrogel. HA increases water uptake into the lens when incorporated into the hydrogel matrix [41,42], which might also have contributed to higher absorption by lens materials with HA. 

It is important to consider the characteristics of soft lenses themselves when selecting a material to manufacture therapeutic lenses. They should still be able to provide clear, comfortable vision, but also be safe to wear for the prescribed period of time. Hydroxyethylmethacrylate (HEMA) is a commonly used conventional lens material but lenses made from this material are limited in their ability to transmit oxygen by their low water content and lens thickness [40]. Silicone hydrogel (SH) soft lens materials were developed relatively recently, in which siloxane moieties were added to the polymer for improved oxygen permeability. SH lenses have the wettability and comfort of conventional hydrogel lenses, but are far better able to meet the oxygen demands of the cornea so that they are safer for extended wear in which the lenses are worn overnight, following eye closure, and may be useful for extended drug release. In addition to altering the release kinetics shown in this work, recent studies have shown that there is reduced protein adsorption on HA-based lenses, which increases wettability and comfort [41,42]. 

## 3. Experimental Section

### 3.1. Solution Preparation

A 3 mg/mL (0.3%) ciprofloxacin solution (Sigma, Oakville, ON, USA) was prepared in Unisol^®^4 (Alcon^®^, Fort-Worth, TX, USA), an unpreserved ophthalmic borate buffer solution. A 0.3% ciprofloxacin solution was used in this study to match commercially available topical ophthalmic ciprofloxacin solutions. The pH was adjusted to approximately pH 4.0 with hydrochloric acid to ensure complete solubility of ciprofloxacin [35]. A 1 mg/mL (0.1%) dexamethasone phosphate solution was also prepared in Unisol^®^4. Linear standard curves were created from the stock drug solution to convert fluorescence readings into concentrations. Amber vials were used throughout the experiment to minimize light exposure to the drugs, which are light sensitive.

### 3.2. Model Materials

2-Hydroxyethyl methacrylate (HEMA), ethylene glycol dimethacrylate (EGDMA) and *N*,*N*-dimethylacrylamide (DMAA) were purchased from Sigma-Aldrich (Oakville, ON, Canada). tris(trimethylsiloxy) silylpropylmethacrylate was purchased from Gelest (Morrisville, PA, USA) and IRGACURE was purchased from CIBA (Mississauga, ON, Canada). The HEMA and TRIS was passed through Aldrich inhibitor removers to remove the polymerizer inhibitor 4-methoxyphenol (MEHQ). All other reagents were used as received. Three soft hydrogel materials, poly-HEMA (pHEMA), pHEMA TRIS and DMAA TRIS were prepared with and without covalent incorporation of HA of molecular weight (MW) 35 or 132 kDa as previously described. Briefly, after passing through the inhibitor removers, 3.6 g of HEMA was mixed with 0.4 g TRIS (90:10 HEMA:TRIS ratio) and 0.2 g of EGDMA. 0.02 g of IRGACURE was added, and the solution was mixed before being cured for 20 minutes in a UV Chamber (CureZone 2 Cont-Trol-Cure) in aluminum molds. DMAA TRIS materials were made in a similar fashion, but with 2.0 g of DMAA added to 2.0 g of TRIS (50:50 DMAA:TRIS ratio), with the weight of the other reagents and the curing process the same. HA was methacrylated and incorporated into the hydrogel materials during UV polymerization. Hydrogel discs were punched out from a sheet of material. The discs had a uniform diameter of 5 mm and a dry weight of 0.034 g +/− 0.006 g, and varied in thickness from 0.93 mm to 1.84 mm. 

### 3.3. Drug Loading

At room temperature, three discs of a given model hydrogel type were rehydrated in Unisol^®^4 for 24 h and then placed in a solution of 3 mg/mL ciprofloxacin or 1 mg/mL dexamethasone phosphate for 24 h.

### 3.4. Release Kinetics

The lens discs from the uptake phase were transferred into Unisol^®^4 after surface residual drug solution was removed with a brief rinse in Unisol^®^4. To approximate in-eye conditions, the lens discs were incubated at 34 °C in a shaking water bath rotating at 100 RPM and released into phosphate buffered saline (pH 7.4). Aliquots of the ciprofloxacin solution were removed at specified time points and measured on a Hitachi F-4500 Fluorescence Spectrophotometer (Hitachi Ltd., Tokyo, Japan) at 274 nm excitation and 419 nm emission wavelengths. Aliquots of the dexamethasone solution were removed at specified time points and measured on a Hitachi UV-2010 spectrophotometer (Hitachi Ltd., Tokyo, Japan) at an absorbance of 241 nm.

### 3.5. Analysis

Data analysis was conducted using a repeat measures ANOVA with time as a within-subject effect and lens material as a between subject factor, and *posthoc* analysis was done using Tukey HSD (Statistica Ver8, Statsoft Inc., Tulsa, OK, USA). A significance level of *p* < 0.05 was considered significantly different. 

### 3.6. Photographs

Photographs of the lens discs were taken using a Canon PowerShot A640 digital camera under natural lighting to show the presence of white precipitates on their surfaces.

## 4. Conclusions

Hyaluronic-acid containing materials may be an advantageous vehicle in drug delivery, particularly for more hydrophobic drugs. Simply swelling the HA modified lens materials in pharmacologically relevant solutions of the drug increased the amount of drug released and resulted in extended release that continued for up to 6 days. Although the silicone hydrogel materials released less ciprofloxacin and dexamethasone phosphate than the conventional hydrogel materials, release amounts were sufficient to be effective against common susceptible and resistant ocular pathogens or to suppress inflammation and the amount released could likely be tailored by changing uptake kinetics or solution concentrations. Ciprofloxacin precipitation on the silicone containing materials suggests that the hydrophobic character of these materials however may limit their potential use in ophthalmic applications. 
